# DeepCRISTL: deep transfer learning to predict CRISPR/Cas9 on-target editing efficiency in specific cellular contexts

**DOI:** 10.1093/bioinformatics/btae481

**Published:** 2024-07-29

**Authors:** Shai Elkayam, Ido Tziony, Yaron Orenstein

**Affiliations:** School of Electrical and Computer Engineering, Ben-Gurion University of the Negev, Beer-Sheva 8410501, Israel; Department of Computer Science, Bar-Ilan University, Ramat Gan 5290002, Israel; Department of Computer Science, Bar-Ilan University, Ramat Gan 5290002, Israel; The Mina and Everard Goodman Faculty of Life Sciences, Bar-Ilan University, Ramat Gan 5290002, Israel

## Abstract

**Motivation:**

CRISPR/Cas9 technology has been revolutionizing the field of gene editing. Guide RNAs (gRNAs) enable Cas9 proteins to target specific genomic loci for editing. However, editing efficiency varies between gRNAs and so computational methods were developed to predict editing efficiency for any gRNA of interest. High-throughput datasets of Cas9 editing efficiencies were produced to train machine-learning models to predict editing efficiency. However, these high-throughput datasets have a low correlation with functional and endogenous datasets, which are too small to train accurate machine-learning models on.

**Results:**

We developed DeepCRISTL, a deep-learning model to predict the editing efficiency in a specific cellular context. DeepCRISTL takes advantage of high-throughput datasets to learn general patterns of gRNA editing efficiency and then fine-tunes the model on functional or endogenous data to fit a specific cellular context. We tested two state-of-the-art models trained on high-throughput datasets for editing efficiency prediction, our newly improved DeepHF and CRISPRon, combined with various transfer-learning approaches. The combination of CRISPRon and fine-tuning all model weights was the overall best performer. DeepCRISTL outperformed state-of-the-art methods in predicting editing efficiency in a specific cellular context on functional and endogenous datasets. Using saliency maps, we identified and compared the important features learned by DeepCRISTL across cellular contexts. We believe DeepCRISTL will improve prediction performance in many other CRISPR/Cas9 editing contexts by leveraging transfer learning to utilize both high-throughput datasets and smaller and more biologically relevant datasets.

**Availability and implementation:**

DeepCRISTL is available via https://github.com/OrensteinLab/DeepCRISTL.

## 1 Introduction

CRISPR/Cas9 technology has been the leading gene-editing technology for more than a decade (Cui *et al.* 2018). By using CRISPR technology, scientists can target a specific DNA sequence with high editing efficiency. The targeting is done via a guide RNA (gRNA) sequence complementary to the target DNA sequence. When choosing the gRNA sequence, there are two main factors to consider: on-target editing efficiency, which is the editing probability, and off-target sites, which reflect the probability of editing at off-target loci. Thus, knowing the on-target editing efficiency and off-target sites is critical for successful editing experiments. As a result, many computational methods were developed to predict the on-target editing efficiency of a given gRNA, most of them are based on machine-learning models trained on experimental editing datasets (Konstantakos *et al.* 2022).

Gene-editing measurements by CRISPR/Cas9 have been produced by various experimental protocols (Zhou *et al.* 2014). High-throughput datasets measure gene editing by lentivirus insertion of the target DNA sequence. While this enables the collection of tens of thousands of measurements, the editing is measured in a synthetic genomic context, which leads to measurements with low correlation to functional and endogenous editing. Functional datasets measure gene editing by observing editing by-products, such as cell livelihood (Haeussler *et al.* 2016). While these datasets produce a signal that is closer to endogenous editing, they are limited to hundreds or thousands of examples, making it difficult to use them to train accurate machine-learning models. Endogenous gene-editing experiments produce the data most biologically relevant, but an experiment is required to produce a measurement for each gRNA (Leenay *et al.* 2019), and as a result, endogenous datasets are too small (up to hundreds) to train machine-learning models. Hence, the main challenge arising from these datasets is how to utilize the high-throughput datasets for the task of functional or endogenous gene-editing prediction.

In recent years, deep neural networks have been revolutionizing the machine-learning field with the availability of abundant datasets and increased efficiency in computational power (Barshai *et al.* 2020). This revolution has sparked great interest and a body of applications in the bioinformatics domain. Deep neural networks were successfully applied in predicting CRISPR/Cas9 on-target editing efficiencies (Wang *et al.* 2020). Seq-deepCpf1 is the first study to use deep learning to solve the challenge of predicting on-target editing efficiencies based on CRISPR high-throughput data (Kim *et al.* 2018). In subsequent studies, Seq-deepCpf1 was outperformed by other deep-learning models, such as DeepHF (Wang *et al.* 2019) and CRISPRon (Xiang *et al.* 2021). Even though these models were trained on large high-throughput datasets and achieved superb performance in cross-validation (e.g. Spearman correlation of 0.87 on a held-out test subset of the DeepHF dataset), their performance on endogenous and functional datasets is much worse (e.g. Spearman correlation < 0.5) (Wang *et al.* 2019).

One of the disadvantages of deep-learning models is their reliance on large datasets for accurate predictions (Barshai *et al.* 2020). Training models with many parameters, as in deep neural networks, may overfit when trained on small datasets. One solution to the problem of training on small datasets is transfer learning (TL) (Tan *et al.* 2018). In TL, a model is trained on a large dataset, which is referred to as the *source* data, and then fine-tuned on a small dataset, which is referred to as the *target* data. In order for TL to improve prediction performance, the source data has to correlate with the target data.

Several TL approaches have been developed, where one of the most popular is the *last-layer* approach. In the last-layer approach, only the weights of the last hidden layer are re-trained in the fine-tuning step. The last-layer approach was applied in the DeepHF study by fine-tuning the model trained on the large DeepHF dataset on each functional dataset to improve the prediction over other functional datasets (Wang *et al.* 2019). The last-layer approach is commonly used in the field of computer vision, where deep models are trained on hundreds of thousands of source data, to transfer trained models with many parameters to fit the target data. While the last-layer TL approach is well suited for computer vision tasks, it may be sub-optimal for bioinformatics tasks due to the smaller size of the source data and less-parameterized networks (Zhuang *et al.* 2021).

In this study, we present a computational method for CRISPR/Cas9 on-target editing efficiency prediction, called DeepCRISTL, based on TL from CRISPRon. We first improved the DeepHF model by using random ensemble initialization and a multi-task technique to utilize all three enzymes measured in the DeepHF study. We then used our newly improved DeepHF model and the CRISPRon model, which were trained on a high-throughput dataset as the source data, and fine-tuned them on smaller functional or endogenous datasets as the target data. Our evaluations show that the fine-tuned CRISPRon outperforms state-of-the-art methods on almost all tested datasets in predicting on-target editing efficiency in a specific cellular context. By interrogating the trained networks, we discovered that while some nucleotide positions are commonly important in various cellular contexts to achieve high efficiency, other positions vary between them.

## 2 Materials and methods

### 2.1 Data

In TL, there are commonly two datasets: the *source* dataset, generally, a large dataset on which pre-training is performed, and the *target* dataset of interest. In this work, source refers to the dataset on which the network is first trained, and target refers to the dataset on which the network is fine-tuned. Table 1 lists the datasets used in this study and their sizes. All datasets used in this study are publicly available (Haeussler *et al.* 2016, Leenay *et al.* 2019, Wang *et al.* 2019, Xiang *et al.* 2021). We normalized the efficiencies of each dataset to a range of 0–1 by min-max normalization.

**Table 1.** The CRISPR/Cas9 on-target editing efficiency datasets used in this study, including their type (high-throughput, functional, or endogenous), size, species, and cell type.

Dataset	Species	Cell type	Size
**High-throughput**			
*DeepHF WT*	Human	HEK293T	55 604
*DeepHF Esp*	Human	HEK293T	58 167
*DeepHF HF*	Human	HEK293T	56 888
*CRISPRon*	Human	HEK293T	23 902
**Functional-U6 promoter**			
*doench2014-Hs*	Human	MOLM13/NB4/TF1	881
*doench2014-Mm*	Mouse	EL4	951
*xu2015TrainHl60*	Human	HL60	2076
*xu2015TrainKbm7*	Human	KBM7	2076
*doench2016_hg19*	Human	A375	2333
*doench2016plx_hg19*	Human	A375	2333
*chari2015Train293T*	Human	HEK293T	1234
*hart2016-Rpe1Avg*	Human	Rpe	4214
*hart2016-HelaLib2Avg*	Human	Hela	3845
*hart2016-Hct1162lib1Avg*	Human	Hct116	4239
*hart2016-HelaLib1Avg*	Human	Hela	4256
**Functional-T7 promoter**			
*morenoMateos2015*	Zebrafish	1-Cell embryos	1020
**Endogenous**			
*leenay*	Human	T	1656

#### 2.1.1 Source datasets

**Figure 1.**
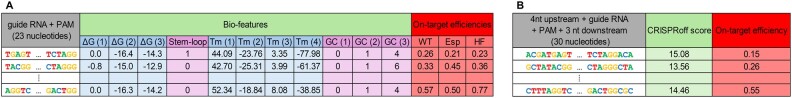
Illustration of the high-throughput datasets used for the pre-training step. (A) DeepHF dataset illustration. Each sequence consists of a 20 nt guide RNA sequence and three PAM nucleotides with GG in the last two positions. Additional 11 sequence-based bio-features are calculated for each sequence. The three right-most columns include the on-target editing efficiencies corresponding to the three enzymes tested in the DeepHF study. (B) CRISPRon dataset illustration. Each sequence consists of four nucleotides upstream, 20 nt guide RNA sequence, three PAM nucleotides, and three nucleotides downstream. One additional feature is calculated by CRISPRoff. The right-most column includes Cas9 on-target editing efficiencies.

We used the DeepHF dataset (Wang *et al.* 2019), which includes on-target editing efficiencies of three enzymes: the wild-type SpCas9, and two types of highly specific SpCas9 variants, eSpCas9 and SpCas9-HF1 (denoted as WT, Esp, and HF, respectively). Each gRNA was measured under the three enzymes, but only gRNAs with read counts >100 were considered valid by the developers of DeepHF and reported in the final dataset. Consequently, few gRNAs do not have on-target editing efficiency measurements for all three enzymes. The dataset includes 55 604, 58 167, and 56 888 gRNAs and their corresponding on-target editing efficiencies per enzyme: WT, Esp, and HF, respectively (Fig. 1A). The combined dataset includes 170 659 on-target editing efficiencies.

We used the CRISPRon dataset which combines two high-throughput datasets: one of 10 592 gRNAs (Xiang *et al.* 2021) and the other of 13 354 gRNAs (Kim *et al.* 2019) totaling 23 902 gRNAs (Fig. 1B). We ran CRISPRoff (Alkan *et al.* 2018), which is part of CRISPRon, to calculate the CRISPRoff score and to add reverse-complement sequences with potential cleavage sites, which resulted in an increase to 25 905 gRNA sequences.

#### 2.1.2 Target datasets

1. Functional: We used the datasets curated by Haeussler et al. (2016). This set includes 18 functional datasets. We filtered out datasets with <500 data points, which left us with 12 datasets overall.
2. Endogenous: We used the dataset produced by Leenay et al. (2019). This dataset contains on-target editing efficiencies of 1656 gRNAs. As far as we know, DeepCRISTL is the only TL-based method to predict on-target editing efficiencies on this dataset.

To avoid train–test leakage, we removed similar gRNAs in the source and target datasets. Since the target datasets are much smaller than the source datasets, which are high-throughput measurements, we removed data from the source datasets. We removed from the DeepHF and CRISPRon datasets all gRNAs that had a gRNA in Hamming distance of <4 over the 23 nt-long gRNA sequence in any of the target datasets. This reduced the DeepHF and CRISPRon datasets from 170 659 to 169 269 combined over the three enzymes and from 25 905 to 22 055 samples, respectively.

### 2.2 Improved DeepHF model architecture

**Figure 2.**
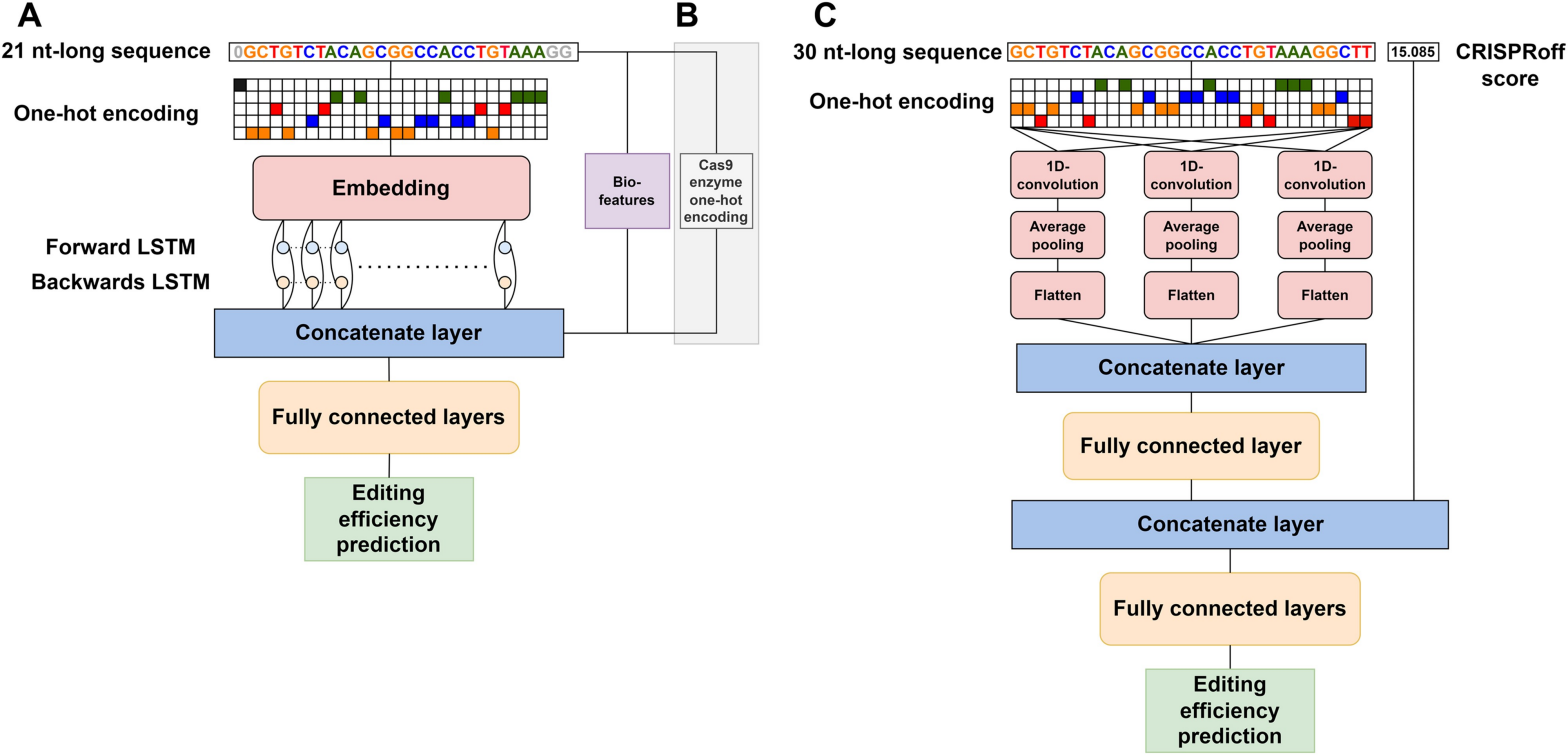
DeepHF and CRISPRon model architectures. (A) DeepHF architecture. The model is comprised of an input layer of a 21 nt-long sequence, which is one-hot-encoded with an additional representation for the start of the sequence, and 11 bio-features. The embedding layer forwards its output to the bi-directional LSTM layer. The LSTM output is being concatenated with the input bio-features, and the concatenated vector is processed through a cascade of fully connected layers to output an on-target editing efficiency prediction. (B) We added three binary inputs for the multi-task DeepHF model to represent the one-hot-encoding of the different Cas9 enzymes. (C) CRISPRon architecture. The model consists of an input layer receiving a 30 nt-long one-hot-encoded sequence. The input is processed through three branches consisting of a 1D-convolutional layer followed by pooling and flattening layers. The concatenated output of the three branches is passed to a fully connected layer and is then concatenated to the CRISPRoff score. The concatenated vector passes through a cascade of fully connected layers to output an on-target editing efficiency prediction.

We developed improved-DeepHF-<WT/Esp/HF> models based on the architecture of the DeepHF model (Wang *et al.* 2019) (Fig. 2A). The DeepHF model combines an embedding layer for vectorizing the nucleotide one-hot-encoded representation, and a bi-directional long-short-term-memory (LSTM) layer for identifying sequence patterns in the gRNA sequence. The bi-directional LSTM is a particular recurrent-neural-network layer, which, unlike standard feed-forward neural networks, has feedback connections making it well-suited for processing and making predictions based on sequence-based data (Barshai *et al.* 2020). Since the last two nucleotides of any gRNA + PAM sequence (occupying in total 23 nucleotides) are GG, the input to the model is the first 21 nucleotides with an additional symbol at the beginning of the sequence to inform the model of the sequence start.

We extended improved-DeepHF-<WT/Esp/HF> model by improved-DeepHF-multi-task model. The multi-task model utilizes the high-throughput datasets of all three enzymes tested in the DeepHF study (Fig. 2B). The model receives an additional input: a one-hot-encoded vector of size three to inform the model which of the three enzyme datasets the specific data point came from (WT, Esp, or HF).

Furthermore, to improve prediction performance we applied the random-ensemble-initialization technique (Sagi and Rokach 2018). We trained 10 identical models on the same datasets but with different random weight initializations. To predict the CRISPR/Cas9 on-target editing efficiency of a gRNA, we calculated the average prediction over all 10 models. We refer to the final multi-task ensemble model as the improved-DeepHF-pre-train model.

### 2.3 Additional input bio-features

To improve prediction performance, the developers of DeepHF added bio-features to the input, which we also included. They showed that adding those bio-features improved prediction performance compared to using the sequence information alone: the Spearman correlations increased from 0.8555, 0.8491, and 0.8512 to 0.8670, 0.8624, and 0.8603 for WT, Esp, and HF enzymes, respectively, when the additional bio-features were added to the recurrent-neural-network architecture (Wang *et al.* 2019). The DeepHF model receives as input 11 bio-features calculated from the gRNA sequence (Fig. 1A). The bio-features include three features of the position accessibility of the secondary structure, one feature of the stem-loop of the secondary structure, four features of the melting temperature, and three features of GC-content information, which is known to be strongly associated with on-target editing efficiency (for more details, see Supplementary Information). The 11 bio-features are concatenated to the LSTM output, which is then passed to the fully connected layer (Fig. 2B). We calculated all bio-features using a script from the DeepHF GitHub repository, which uses the ViennaRNA package (Lorenz *et al.* 2011).

CRISPRon adds one non-sequence feature $\Delta G_{B}$, termed *CRISPRoff score* (Fig. 2C), which is an approximation of the free energy of the Cas9–gRNA–DNA binding by the energy model used in CRISPRoff (Alkan *et al.* 2018). $\Delta G_{B}$ calculation estimates the gRNA–DNA hybridization energy, the energy required to unwind the DNA, and the gRNA free energy with a correction factor for the PAM.

### 2.4 Training, hyper-parameters search, and evaluation

#### 2.4.1 Improved-DeepHF-pre-train model training

We split the reduced DeepHF dataset, which we created to avoid leakage with the target datasets, to training and test sets with sizes of 85% and 15%, respectively. To avoid train–test leakage, we partitioned the gRNAs into clusters, ensuring that any pair of gRNAs with a Hamming distance of <5 across the 23 nucleotides were in the same cluster. Subsequently, we sorted the clusters by size and assigned them to training or test sets while preserving the train–test split ratios of 85% and 15%, respectively. To fairly compare between the single-task models and the multi-task model, we used the same partition to training and test sets in all comparisons. Since some of the gRNAs do not have on-target editing efficiency values for all three enzymes, we constrained the test set to have on-target editing efficiency values for all three enzymes in addition to not having a gRNA in Hamming distance of <5 in the training set. Hence, we could fairly evaluate this test set on both the single-task and multi-task models.

We applied a random hyper-parameter search to find optimal hyper-parameters of the improved-DeepHF models (single-task and multi-task models) with a random 10% of the training set serving as the validation set. The searched hyper-parameters included initial learning rate, batch size, optimizer, the activation function of the last layer, weight initialization, dimensions of the embedding layer, dropout rates of the embedding, LSTM, and the fully connected layers, the number of neurons in each of the fully connected layers and in the LSTM layers, and the number of fully connected layers (Supplementary Table S1). After choosing the optimal hyper-parameters, we trained 10 randomly initialized models and used all 10 in our ensemble model. In each of the training procedures, we applied early stopping on the validation set to avoid over-fitting.

After comparing all four types of improved-DeepHF models (WT, Esp, HF, and multi-task), we selected the improved-DeepHF-multi-task model as our final improved-DeepHF model. To improve prediction performance by the improved-DeepHF model, we trained 10 randomly initialized models on all the dataset (train, validation, and test sets). We refer to this model as improved-DeepHF-pre-train and used it as the pre-trained model for TL from DeepHF.

#### 2.4.2 CRISPRon-pre-train model training

We trained the CRISPRon model using the optimal hyper-parameters reported in the CRISPRon study (Xiang *et al.* 2021): a learning rate of 0.0001, the Adam optimizer, a batch size of 500, and 6 models in the random ensemble initialization (Fig. 2C). We trained CRISPRon on the reduced CRISPRon dataset, which we created to avoid leakage with the target datasets (Supplementary Fig. S1A). We did not test CRISPRon in cross-validation as it was based on the architecture and hyper-parameters from the CRISPRon study.

#### 2.4.3 DeepCRISTL model fine-tuning

The functional and endogenous datasets are much smaller; hence, the choice of the specific test set can greatly affect the evaluated prediction performance. To obtain a robust evaluation of prediction performance, we repeated the evaluation procedure five times, each time using a different partition to 80% training and 20% test sets (Supplementary Fig. S2). To prevent train–test leakage, we partitioned the gRNAs into clusters, ensuring that any pair of gRNAs with a Hamming distance of at most 4 across the 23 nucleotides were in the same cluster. Then, we assigned them to training and test sets in a random order while preserving the desired set size ratio.

To determine the maximum number of epochs for the improved-DeepHF-pre-train model, we applied 10-fold cross-validation over the training set. In each of the 10 iterations, we found the optimal number of epochs by early stopping. Then, we set the maximum number of epochs over the whole training set as the rounded average over the 10 optimal numbers of epochs. After setting the maximum number of epochs, we combined the training and validation sets in order to train on 80% of the data and evaluate prediction performance on the test set. In each of the 5 iterations, we fine-tuned the 10 randomly initialized pre-trained models, and finally used all 10 as our TL ensemble model. For the CRISPRon model, we used the same approach as in the CRISPRon study: we utilized early stopping with a patience parameter set to 100 epochs and a maximum of 5000 epochs (Supplementary Fig. S1B).

### 2.5 Transfer-learning approaches

**Figure 3.**
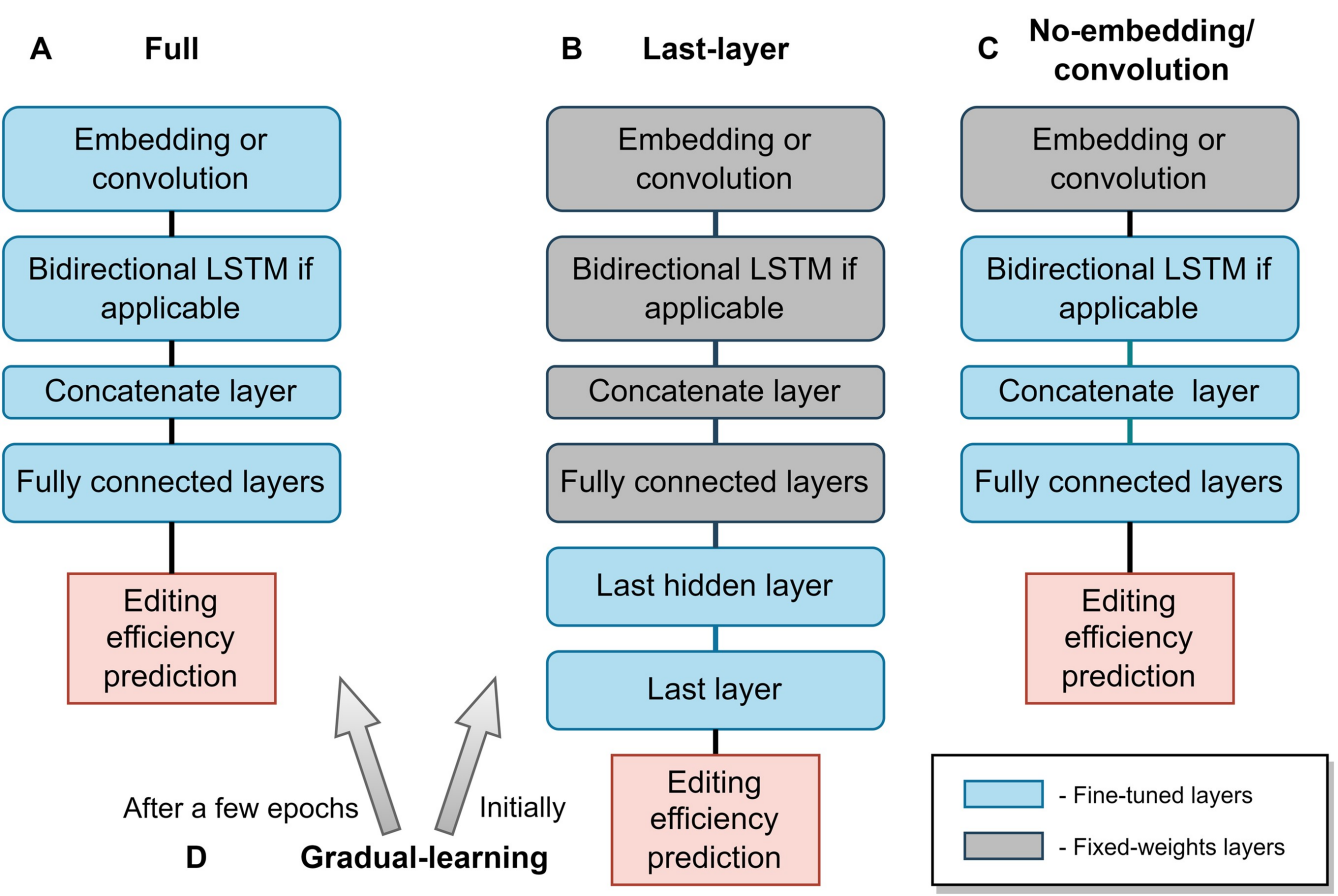
The transfer-learning approaches compared in this study. (A) The full approach fine-tunes all model weights. (B) The last-layer approach fine-tunes only the weights of the last hidden layer and the output layer. (C) The no-embedding/convolution approach fine-tunes all model weights except for the weights of the embedding or the convolutional layer. (D) The gradual-learning approach first fine-tunes only the weights of the last hidden layer and the output layer and then continues to fine-tune all model weights with a smaller learning rate.

On each endogenous and functional dataset, representing different cellular contexts, we tested four types of TL approaches (Fig. 3):

1. Full: Fine-tuning is performed on all model weights.
2. Last-layer: Fine-tuning is performed only on the weights of the last hidden layer and the output layer.
3. Gradual-learning: Initially, fine-tuning is performed on the weights of the last hidden layer with the original learning rate. Then, fine-tuning continues on all model weights with a smaller learning rate.
4. No-embedding/convolution: Fine-tuning is performed on all model weights except for the weights of the embedding/convolution layer.

We added two types of trained models for comparison:

1. No TL: Using only the pre-train model, which was trained on the high-throughput dataset.
2. No pre-train: Training the model only on the endogenous or functional dataset, which represents a specific cellular context.

The different TL approaches represent different trade-offs and combinations of fine-tuning the last hidden layer only and training the full model. The gradual-learning approach is a unique combination, which leverages both the pre-trained weights in fine-tuning the last hidden layer and the full model fine-tuning on a more refined scale. The no-embedding/convolution approach is based on the assumption that the embedding/convolution layer models general patterns in the gRNA sequence. Hence, there is no need to fine-tune the weights of that layer for a modified representation of the nucleotides. The different approaches also represent trade-offs in terms of training runtime (Supplementary Table S2).

### 2.6 Interpretability

To gain biological insights behind the mechanism of CRISPR/Cas9 on-target editing for each of the datasets, which represent different cellular contexts, we applied the saliency-map technique [as was previously applied (Lanchantin *et al.* 2017)]. To calculate the saliency map for a specific fine-tuned model or pre-train model, we used the samples it was fine-tuned or pre-trained on, respectively. We calculated the average attribution of each input nucleotide feature (except the GG of the PAM) as the gradient of these features with respect to the output over each of the samples. We assigned these averages as the final attribution scores. For *X_i_*_,_*_j_* and *Y_i_* denoting feature *j* in and the output of sample *i*, respectively, we define by *I_j_* the attribution of feature *j* over *n* samples:

(1) $$I_{j}=\frac{1}{n}\sum_{i=1}^{n}\frac{\partial Y_{i}}{\partial X_{i,j}}$$

Since our DeepCRISTL model is an ensemble model composed of multiple models, we performed this procedure for each nucleotide feature on each model and averaged the attribution for each nucleotide feature over the models. We visualized the sequence preferences learned by the DeepCRISTL models through sequence logos, where the height of each letter within the sequence is proportional to the feature importance derived from the saliency-map technique.

## 3 Results

### 3.1 Our newly improved DeepHF model

We developed the improved-DeepHF-pre-train model, a modified DeepHF model and training scheme, to improve on-target editing efficiency prediction. The original DeepHF study reported the Pearson correlation of on-target editing efficiencies between the three tested enzymes. All enzyme pair-wise on-target editing efficiency Pearson correlations were between 0.6 and 0.8. Thus, to benefit from the combined correlation as shared feature information, we trained a multi-task version of the model on all three enzymes together. In addition, we utilized a random-ensemble-initialization technique to increase prediction performance and robustness. We gauged prediction performance by Spearman correlation of predicted and measured on-target editing efficiencies on a held-out test set of 15% of the DeepHF dataset, as was previously done in the original DeepHF study (Wang *et al.* 2019), while ensuring that any pair of gRNAs in the test and training sets are in Hamming distance of at least 5 over the 23 nt-long gRNA sequences.

**Figure 4.**
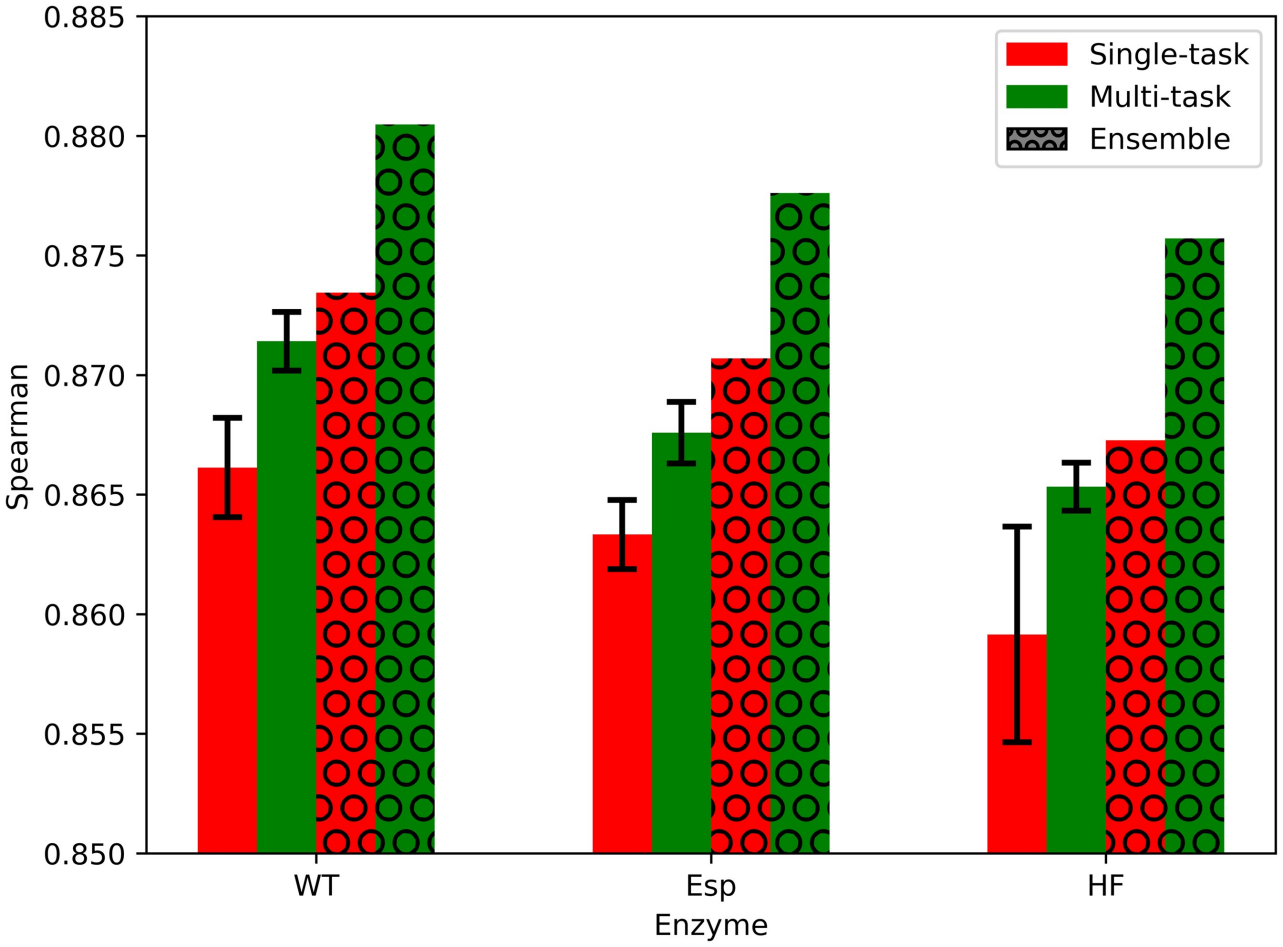
Prediction performance of our newly improved DeepHF model over the three high-throughput datasets (WT, Esp, HF). Four model variants are compared: single-task for one enzyme, multi-task for all enzymes, and with and without the random-initialization-ensemble technique.

Our multi-task model improved prediction performance over the high-throughput datasets of all three enzymes (Fig. 4). The multi-task version achieved an average Spearman correlation of 0.871, 0.868, and 0.865 in cross-validation on the DeepHF dataset compared to the single-task version, which achieved 0.866, 0.863, and 0.859 for the WT, Esp, and HF enzymes, respectively (*P*-values < 0.001 over 10 differently initialized models, Wilcoxon rank-sum test). This shows the power gained by combining correlated datasets into a single multi-task model.

The addition of the random-ensemble-initialization technique using 10 differently initialized models improved prediction performance further (Fig. 4). The ensemble of randomly initialized multi-task models achieved a Spearman correlation of 0.880, 0.878, and 0.876 compared to the single multi-task model, which achieved 0.871, 0.868, and 0.865 for WT, Esp, and HF enzymes, respectively. When testing other numbers of randomly initialized models, we observed no significant improvement over 10 models at the cost of an increase in training runtime (Supplementary Fig. S3). Thus, we chose 10 as the number of randomly initialized models in the ensemble of the improved-DeepHF-pre-train model.

### 3.2 Evaluation of transfer-learning approaches

Once we established that the improved-DeepHF-pre-train model was outperforming the original DeepHF model, we turned to fine-tuning it and the CRISPRon-pre-train model on functional and endogenous datasets to predict the on-target editing efficiency in a specific cellular context. We compared four types of TL approaches: last-layer, no-embedding/convolution, full, and gradual-learning, and two baseline models: no TL and no pre-train, to choose the best TL approach for the task of on-target editing efficiency prediction in a specific cellular context. We tested the different approaches in cross-validation on the various datasets. Over each dataset, we evaluated on 5 held-out test sets of 20% of the data each. We gauged prediction performance by Spearman correlation of measured and predicted on-target editing efficiencies.

**Figure 5.**
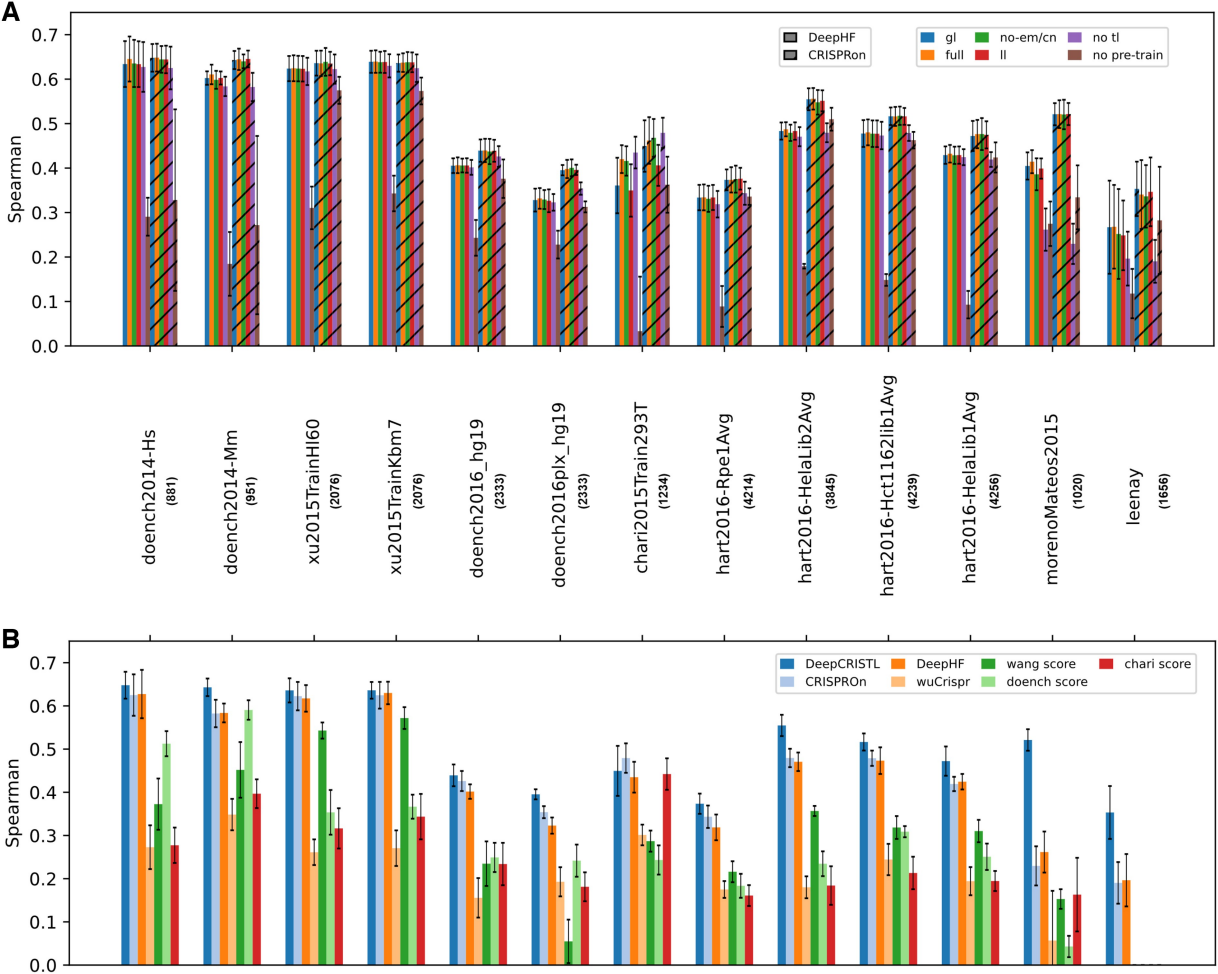
Comparison of various TL approaches and state-of-the-art methods in predicting on-target editing efficiencies in specific cellular contexts. Prediction performance was gauged by average Spearman correlation of predicted and measured on-target editing efficiencies over five held-out test sets of 20% of the data each. (A) Both improved-DeepHF- and CRISPRon-pre-train models were used for the TL approaches comparison. tl: transfer learning; gl: gradual learning; em/cn: embedding/convolution; ll: last layer. (B) Comparison of DeepCRISTL and state-of-the-art methods in predicting on-target editing efficiencies in specific cellular contexts. The average Spearman correlation over five held-out test sets is reported for each dataset. The leenay dataset has scores only for DeepCRISTL, improved-DeepHF, and CRISPRon models since the predictions by other the methods on this dataset were unavailable in the Haussler *et al.* study.

The comparison of various TL approaches combined with the improved-DeepHF- or CRISPRon-pre-train models shows that fine-tuning all model weights performed the best on average for both models (*P*-values < 0.024 comparing CRISPRon with full TL approach to non-TL approaches over 5 held-out test sets) and that all TL approaches combined with CRISPRon achieved better prediction performance compared to all TL approaches combined with improved-DeepHF (Fig. 5A). The TL approaches performed similarly. For example, the no-convolution-TL approach combined with CRISPRon achieved an average Spearman correlation of 0.5177 on the hart2016-Hct1162lib1Avg dataset, compared to 0.5165, 0.5162, 0.5163 of gradual-learning, full, and last-layer TL approaches, respectively (*P*-values > 0.06, Wilcoxon-rank-sum test). On one dataset, chari2015Train293T, the no-TL model performed better than the TL approaches, and both outperformed the last-layer approach and no-pre-train model. To conclude, we chose full-TL as the approach to train our final DeepCRISTL models combined with the CRISPRon-pre-trained model. The complete results are in Supplementary Table S3.

### 3.3 Comparison of DeepCRISTL to existing methods in predicting on-target editing efficiency in a specific cellular context

To gauge the ability of DeepCRISTL and existing methods to predict CRISPR/Cas9 on-target editing efficiencies in specific cellular contexts, we compared the Spearman correlation achieved by various state-of-the-art methods on all available functional datasets. For each dataset, we held out a test set of 20% of the data to evaluate prediction performance on it. We report the average over five such test sets for each dataset. We excluded the endogenous dataset of Leenay *et al.* since its predicted scores by all other methods mentioned in the Haussler *et al.* study were not available as part of that study (Haeussler *et al.* 2016).

DeepCRISTL outperformed all other methods in on-target editing efficiency prediction on average and in all but one cellular context (Fig. 5B) (*P*-values < 0.002 compared to other methods over the 12 functional datasets). For example, DeepCRISTL achieved an average Spearman correlation of 0.556 on the hart2016-HelaLib2Avg dataset, while the second-best and third-best were CRISPRon and improved-DeepHF, which achieved an average Spearman correlation of 0.479 and 0.470 (*P*-values < 0.032 over 5 held-out test sets, Wilcoxon-rank-sum test), respectively. The largest improvement was achieved on the morenoMateos2015 and leenay datasets: an average Spearman correlation of 0.521 and 0.340 compared to 0.261 and 0.196 of the second best (*P*-values < 0.032, Wilcoxon-rank-sum test), respectively. This is likely due to the low correlation of the high-throughput datasets with morenoMateos2015 and leenay datasets. The complete results are in Supplementary Table S3.

### 3.4 Cross-prediction performance over different cellular contexts

Next, we aimed to evaluate the prediction performance of each DeepCRISTL model across the various cellular contexts to gauge the generalizability of the models. To this end, we evaluated the prediction performance of each fine-tuned model and the CRISPRon-pre-train model on each target dataset, where we used the complete datasets in this evaluation.

**Figure 6.**
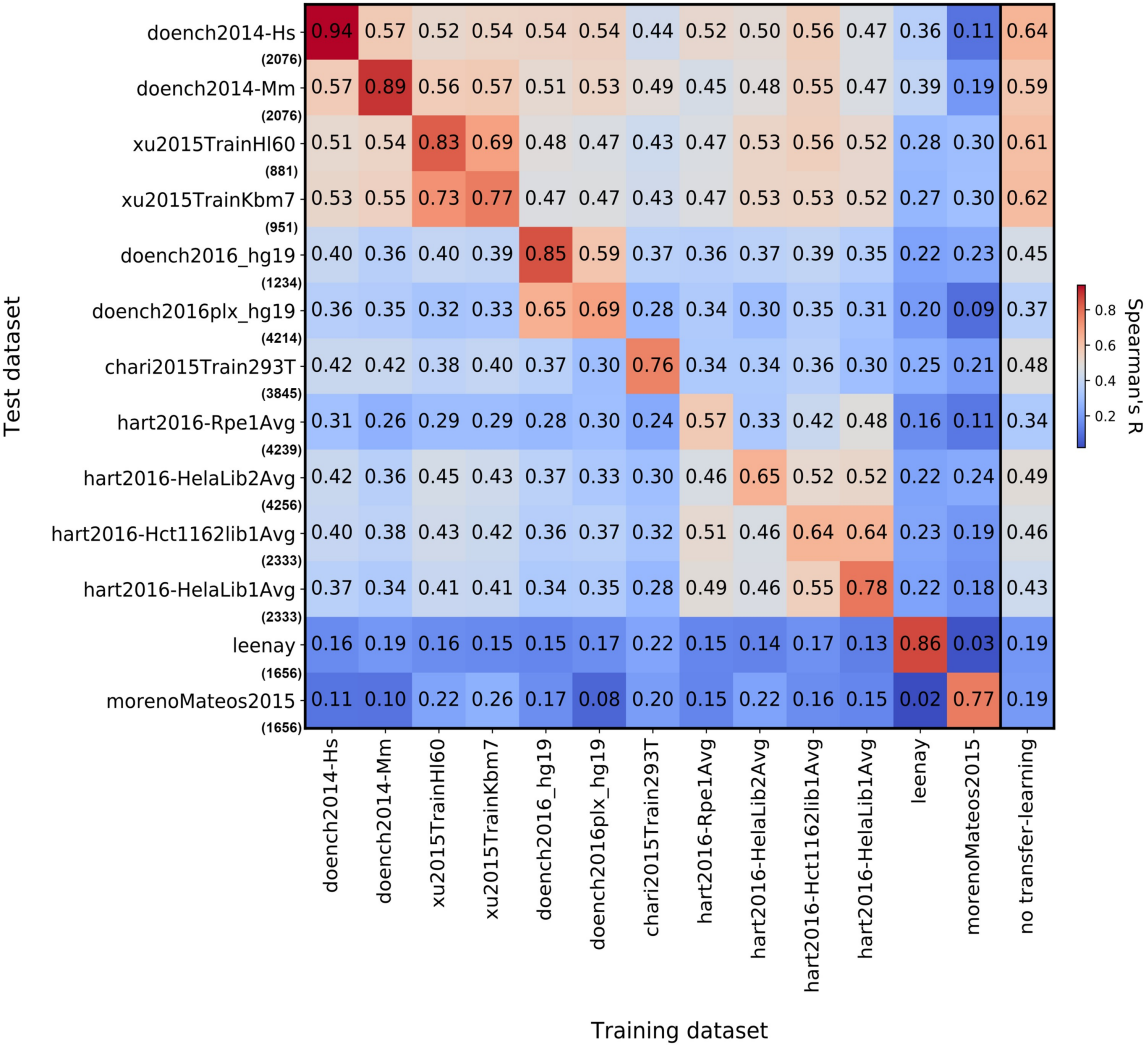
Prediction performance of the DeepCRISTL models and the CRISPRon-pre-train model on each target dataset. Dataset name on *x*-axis indicates the dataset the model was fine-tuned on. Dataset name and size on *y*-axis indicate the test set.

The results show the similarities and differences among the cellular contexts and experimental techniques (Fig. 6). For example, the xu2015TrainHl60 and xu2015TrainKbm7 models achieved a Spearman correlation of 0.69 and 0.73 on each other, respectively. Similarly, the doench2016 models performed better on each other compared to other datasets, as well as the hart models. Most strikingly, all models performed much worse on the leenay and morenoMateos2015 datasets, hinting at the unique characteristics of the cellular context and/or experimental technique they were measured in. Last, we observed that the CRISPRon-pre-train model performed better than most fine-tuned models on datasets they were not fine-tuned on. Thus, we conclude that when no target data is available, it is advisable to use CRISPRon rather than DeepCRISTL, and when target data from the same or similar cellular context is available, it is advisable to use a DeepCRISTL model, which was fine-tuned to that, or if unavailable, similar cellular context.

### 3.5 Visualization of gRNA sequence preferences

To gain insights into the mechanism of gRNA on-target editing efficiency sequence preferences in various cellular contexts, we visualized the sequence preferences learned by DeepCRISTL as sequence logos. We generated nucleotide importance scores using the saliency-map technique for each of the fine-tuned models (each corresponding to a different cellular context) (Lanchantin *et al.* 2017). We then plotted each letter in that sequence with its height being its importance score. We also generated the sequence preference of the CRISPRon-pre-train model to compare the results before and after TL.

**Figure 7.**
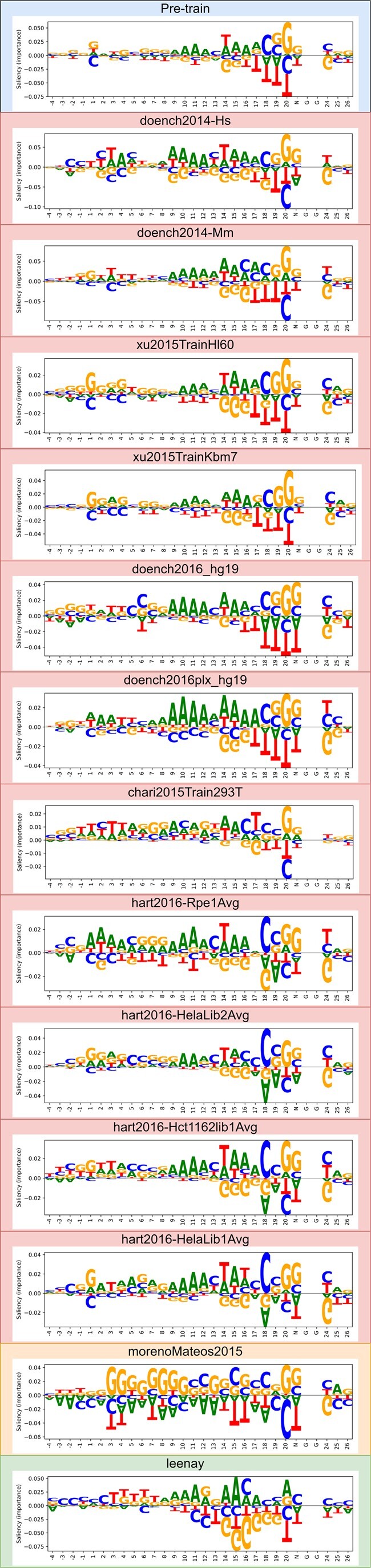
DeepCRISTL model interpretation. We used the saliency-map technique to generate a sequence logo for each of the fine-tuned models, which represents the on-target sequence preferences learned in each dataset.

Figure 7 shows the on-target preferences learned by the model in each cellular context. The “G” in position 20 is favored by almost all models, which is consistent with previous findings (Wang *et al.* 2019). Similarly, the stretch of “A”s upstream of “C” in position 18 is favored by almost all models, and “G” downstream of the PAM is disfavored by all models. The most striking difference lies in morenoMateos2015 and leenay preferences. morenoMateos2015 shows an affinity for “G” in almost all positions which could be a result of using a T7 promoter. In the leenay dataset, “A” is preferred in position 20 compared to other datasets in which the highest importance is attributed to “G.” In addition, the most important nucleotide is “A” in position 15 compared to 20 in other datasets. More subtle differences among the U6 datasets lie in the first 10 positions of the guide RNA, showing distinct preferences consistent with the prediction performance categorization (Fig. 6).

## 4 Discussion

In this study, we developed a novel method, DeepCRISTL, to predict Cas9 on-target editing efficiencies in specific cellular contexts based on TL from high-throughput datasets. We fine-tuned CRISPRon on smaller endogenous or functional datasets, which represent different cellular contexts. The resulting DeepCRISTL model achieved state-of-the-art prediction performance on held-out test sets of the same datasets. To interpret the trained models, we applied the saliency-map technique to visualize the principles the model has learned before and after fine-tuning. By inspecting the generated sequences logos, we observed key positions that are shared among most datasets. Other positions were generally unique, implying that there may be specific cellular factors involved in the on-target editing process.

A key feature of DeepCRISTL improved performance compared to existing methods for on-target editing efficiency prediction in a specific cellular context is the full-TL approach, which is used to fine-tune the pre-trained model weights. This approach leads to state-of-the-art performance in each cellular context. In contrast to existing methods that applied TL by fine-tuning only the last hidden layer of the model, DeepCRISTL fine-tunes all model weights. The full-TL approach was also superior to the last-layer approach in our recent DeepZF method (Aizenshtein-Gazit and Orenstein 2022), which may hint on its superiority in bioinformatic applications where the networks are less parametrized and less data is available for the fine-tuning step compared to other machine-learning domains. We expect that as new high-throughput datasets will be produced in greater scale, prediction performance in specific cellular contexts will further improve using full-TL and similar TL approaches (Supplementary Fig. S4).

Recently, Corsi *et al.* have demonstrated that DeepCRISTL does not outperform competing methods when tested on datasets other than the one it was fine-tuned on (Corsi *et al.* 2023). Indeed, we developed DeepCRISTL to succeed in the specific cellular context it was trained on. As such, the specific fine-tuned model is not aimed to perform well on other cellular contexts. The fine-tuning of bioinformatics methods to specific cell types or cellular contexts is very common (Keilwagen *et al.* 2019), and we demonstrated its successful application to the on-target editing efficiency prediction task by DeepCRISTL and its unique TL approach. If no data is available for the specific or similar cellular context, we recommend using CRISPRon, as our results show that CRISPRon outperforms all other non-fine-tuned methods and DeepCRISTL models fine-tuned on other cellular contexts.

There are several aspects that require further research in the future. First, further improvement may be achieved by including additional bio-features. While DeepHF uses 11 bio-features, CRISPRon uses only one. Adding the bio-features defined by DeepHF or other studies to CRISPRon may improve its prediction performance, and as a result the performance of DeepCRISTL fine-tuned models. Second, combining epigenetic markers, such as DNA methylation and open chromatin, may improve prediction performance (Chuai *et al.* 2018). Since epigenetic data is not always available, there is still room for sequence-only-based methods or using predicted epigenetic markers (Schreiber *et al.* 2020). A key challenge in this aspect is how to expand the input of the pre-trained model, which is based on high-throughput data, which lacks the cellular and genomic contexts, by additional cellular information while enabling efficient optimization of the new and previously trained model weights. Third, an additional improvement can be achieved by combining all functional datasets into one dataset and using it to fine-tune the model as an intermediary step before the final fine-tuning stage. This may be highly challenging as different cell types and species may be too distinct to be easily merged. One way of combining datasets can be achieved by linear scaling of the on-target editing efficiencies as was done in the CRISPRon study (Xiang *et al.* 2021). Fourth, we plan to improve CRISPR/Cas9 off-target prediction in specific cellular contexts using similar TL techniques as we used in this study (Kota *et al.* 2021, Yaish *et al.* 2022, Yaish and Orenstein 2024). While the underlying molecular process is the same in on- and off-target editing, data characteristic are different and include highly imbalanced datasets and fewer informative tagged examples in the off-target case. Last, we plan to make DeepCRISTL easy to use for biologists by developing a web-server that will receive as input a gRNA sequence and predict its on-target editing efficiencies in different cellular contexts.

## 5 Conclusion

We developed a new method, DeepCRISTL, to predict the on-target editing efficiencies of CRISPR/Cas9 given a gRNA and PAM sequence. DeepCRISTL’s unique approach utilizes the state-of-the-art CRISPRon model with full-TL approach for TL. DeepCRISTL outperforms the state-of-the-art in on-target editing efficiency prediction, and its learned preferences are biologically relevant. We hope to see DeepCRISTL used to predict on-target editing efficiencies of functional and endogenous experiments, and aspire after similar developments for experimental datasets based on high-throughput sequencing in other biological domains.

## Supplementary Material

btae481_Supplementary_Data

## Data Availability

Our fine-tuned models are available as part of our Github repository at https://github.com/OrensteinLab/DeepCRISTL. The datasets used are all available and are in the public domain. DeepHF: https://www.nature.com/articles/s41467-019-12281-8 - supplementary data 2. CRISPRon: https://www.nature.com/articles/s41467-021-23576-0 - supplementary data 1. Kim: https://www.science.org/doi/10.1126/sciadv.aax9249 - supplementary table 1. Haeussler: https://genomebiology.biomedcentral.com/articles/10.1186/s13059-016-1012-2 - additional file 14. Leenay: https://figshare.com/articles/dataset/Analyzed_T-Cell_DNA_Repair_Outcomes/6957125?file=12860156.
