# Supplementary material for: DeepCRISTL: deep transfer learning to predict CRISPR/Cas9 on-target editing efficiency in specific cellular contexts
Source: Bioinformatics. 2024 Jul 29;40(8):btae481. doi: 10.1093/bioinformatics/btae481 (PMC11319645; doi:10.1093/bioinformatics/btae481)
Supplement: btae481_Supplementary_Data [file btae481_supplementary_data.zip › DeepCRISTL___correction___final_supp.pdf]

Supplemental Information
DeepCRISTL: Deep transfer learning to predict CRISPR/Cas9 on-target editing efficiency in specific cellular contexts

Shai Elkayam [1,†], Ido Tziony [2,†], and Yaron Orenstein [2,3]
[1]*School of Electrical and Computer Engineering, Ben-Gurion University of the Negev, Beer-Sheva 8410501, Israel.* [2]*Department of Computer Science, Bar-Ilan University, Ramat Gan, 5290002, Israel.* [3]*The Mina and Everard Goodman Faculty of Life Sciences, Bar-Ilan University, Ramat Gan, 5290002, Israel.* [†]*Equal contribution.*

I. DESCRIPTION OF ADDITIONAL BIO-FEATURES

We used four types of additional bio-features in the improved-DeepHF model:

1. RNA secondary structure free energy - dG is the free energy of the predicted secondary structure.
   (a) dG binding 20: The free energy of the gRNA bound structure.
   (b) dG binding 7 to 20: The free energy of the gRNA bound structure over nucleotides 7 to 20.
   (c) dG: The free energy of the gRNA and the PAM first nucleotide bound structure.
2. Stem-loop - a binary feature that indicates whether a stem-loop structure is predicted to form or not.
3. Melting temperature - the melting temperature is related to the free energy of the gRNA.
   (a) Tm global: Melting temperature of the entire gRNA sequence.
   (b) 4mer start: Melting temperature of nucleotides 1 to 4.
   (c) 8mer middle: Melting temperature of nucleotides 5 to 12.
   (d) 5mer end: Melting temperature of nucleotides 16 to 20.
4. GC-content - the bio-feature represents the number of guanine (G) and cytosine (C) nucleotides in the gRNA sequence.
   (a) GC $>$ 10: A binary feature that indicates whether there are more than 10 G and C nucleotides.
   (b) GC $<$ 10: A binary feature that indicates whether there are less than 10 G and C nucleotides.
   (c) GC count: The number of G and C nucleotides.

We used one additional feature in the CRISPRon model, termed *CRISPRoff* score: ΔG_B, which is computed using the energy function in the CRISPRoff pipeline.

Hyper-parameter search space		
**Hyper parameter**	**Search space**	**Comment**
Last activation	sigmoid, linear	The activation function of the last fully connected layer
Initializer	he_uniform, lecun_uniform, normal, he_normal	Distribution of the initializer function
Batch size	30 + 10i , i={0,1,...,17}	
Optimizer	Nadam, SGD, RMSprop, Adagrad, Adadelta, Adam, Adamax	
Embedding dimension	30 + i , i={0,1,...,50}	
Embedding dropout rate	0.1 + 0.1i , i={0,1,...,8}	
LSTM cell units	50 + 10i , i={0,1,...,18}	
LSTM dropout rate	0.1 + 0.1i , i={0,1,...,8}	
Recurrent LSTM dropout rate	0.1 + 0.1i , i={0,1,...,8}	
Number of fully connected (FC) layers	1, 2, 3, 4, 5	
Number of units in FC layers	1, 2, 3, 4, 5	
FC dropout rate	0.1 + 0.1i , i={0,1,...,8}	

Supplementary Table I: Detailed hyper-parameters search space for the improved-DeepHF model. All hyper-parameters have been randomized from a pre-defined range or from a pre-defined array using a uniform distribution.

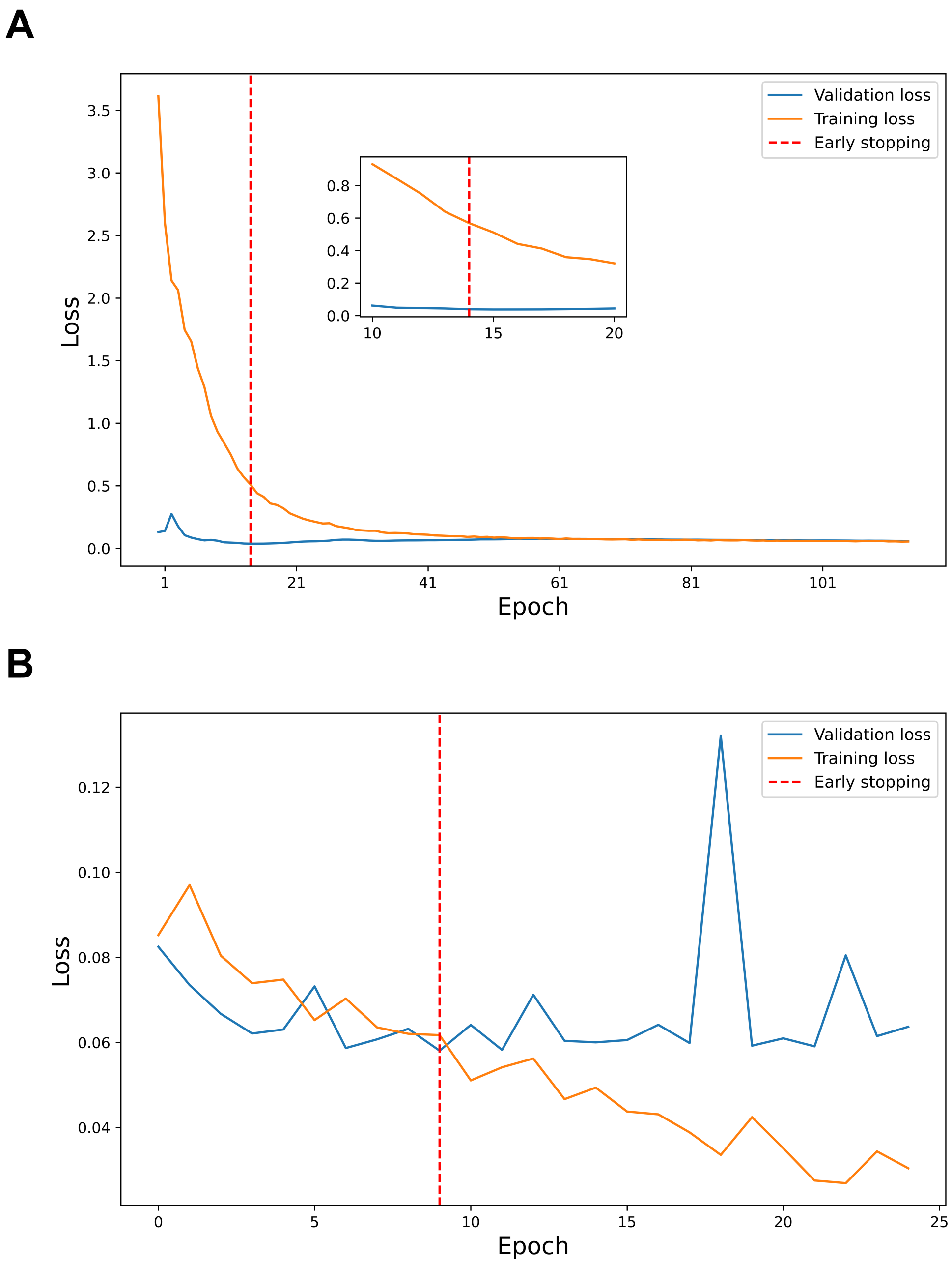

Supplementary Figure 1: Train-validation loss curves of pre-training and fine-tuning one of the models of DeepCRISTL ensemble. (A) CRISPRon-pre-train model validation on 20% of the CRISPRon dataset. Loss values reported starting from the first epoch. (B) Full transfer learning of the CRISPRon-pre-train model on morenoMateos2015 dataset. Loss values reported from the pre-trained model.

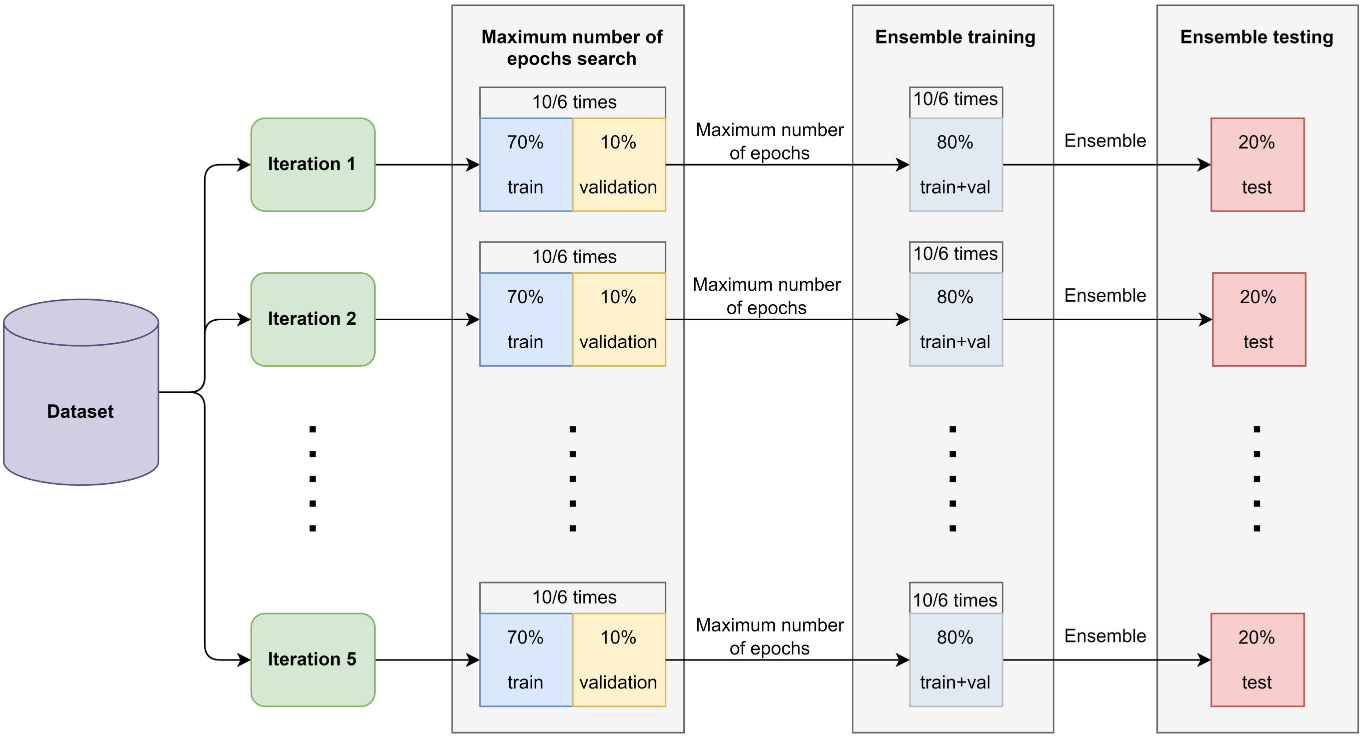

Supplementary Figure 2: Data partitioning for training, hyper-parameter search, and model evaluation. We partitioned the complete dataset in each of the 5 iterations. In each iteration, we split each dataset into training, validation, and test sets of size 0.7, 0.1, and 0.2 of the dataset, respectively, using our partitioning approach which ensures Hamming distance greater than 4 between train and validation to test set gRNAs. We then trained 10 or 6 models (for DeepHF- or CRISPRon-pre-train models, respectively) and computed the rounded average of the number of epochs at which the early stopping occured over the training of each model. We then trained on the training and validation sets combined using the average as the maximum number of epochs in the early stopping. Last, we trained 10 or 6 models as part of the random ensemble initialization (for DeepHF- or CRISPRon-pre-train models, respectively).

TL approach	Average runtime (seconds)	Average number of epochs	Comment
full	53.91 ± 4.36	10.2 ± 2.23	
ll	69.23 ± 6.59	11.2 ± 3.54	
gl	41.86 ± 1.31	4.40 ± 0.80	There is a prior step of ll TL.
no-conv	65.49 ± 3.00	10.6 ± 1.62	
no TL	N/A	N/A	No fine-tuning.
no pre-train	70.59 ± 15.6	21.8 ± 10.2	

Supplementary Table II: The training runtime of each TL approach. The measurements are reported over the morenoMateos2015 dataset combined with the CRISPRon-pre-train model. We averaged the runtime and number of epochs over 5 different runs of each TL approach. The runtime includes the hyper-parameters search step. The training was performed on an NVIDIA A100 GPU.

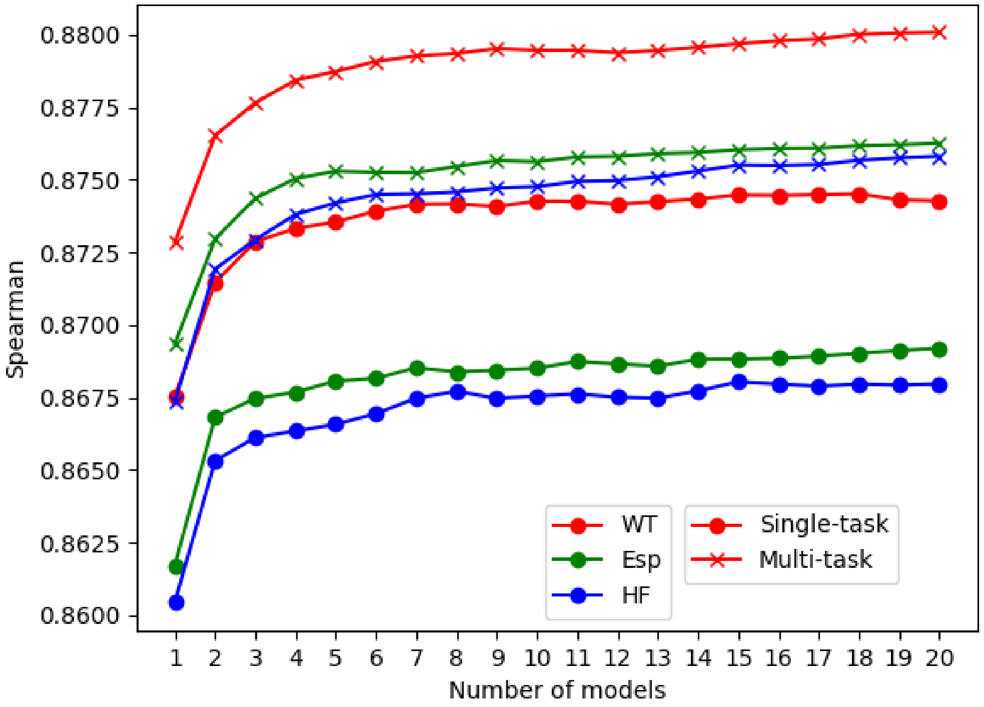

Supplementary Figure 3: Prediction performance as a function of the number of randomly initialized models in the ensemble of improved-DeepHF. We tested the multi-task model on each of the 3 enzymes separately. Spearman correlation was calculated between predicted and measured on-target editing efficiencies.

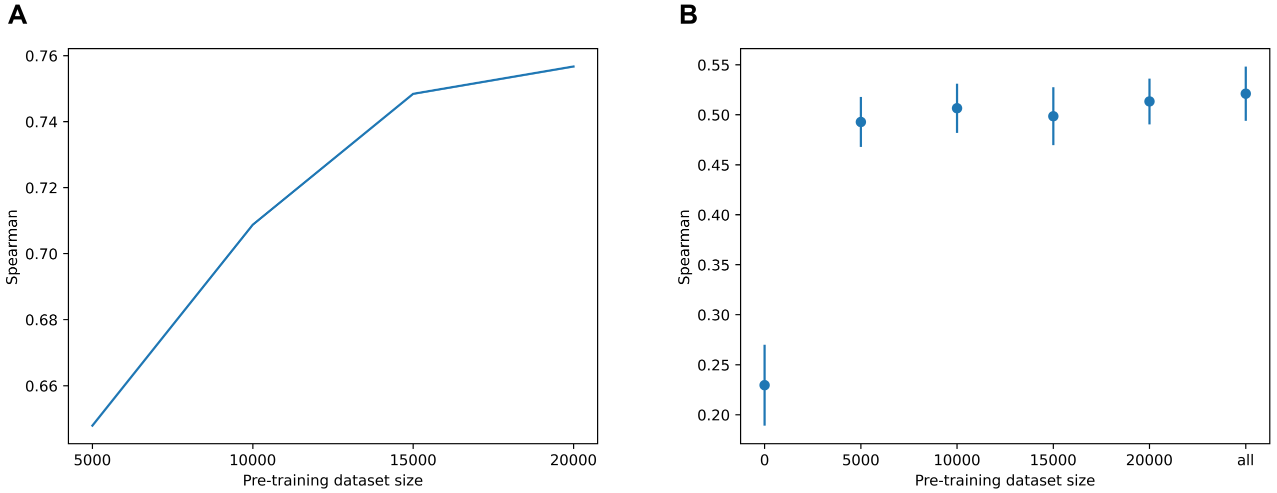

Supplementary Figure 4: Prediction performance as a function of training set size. We pre-trained the CRISPRon model on variable training set sizes. (A) Prediction performance on a held-out subset of 20% of the source dataset. (B) Prediction performance achieved by the full-TL approach on the morenoMateos2015 dataset. The average Spearman is reported over five held-out test sets of 20% each.
